# Oxygen and early animals

**DOI:** 10.7554/eLife.34756

**Published:** 2018-02-06

**Authors:** Kalle T Rytkönen

**Affiliations:** 1 Institute of BiomedicineUniversity of TurkuTurkuFinland; 2 Turku Centre for BiotechnologyUniversity of Turku and Åbo AkademiTurkuFinland

**Keywords:** hypoxia-inducible factor, Tethya wilhelma, oxygen sensing, early animal evolution, Porifera, Ctenophora, Other

## Abstract

The biology of sponges provides clues about how early animals may have dealt with low levels of oxygen.

**Related research article** Mills DB, Francis WR, Vargas S, Larsen M, Elemans CPH, Canfield DE, Wörheide G. 2018. The last common ancestor of animals lacked the HIF pathway and respired in low-oxygen environments. *eLife* **7**:e31776. doi: 10.7554/eLife.31176

Once upon a time – between 800 and 550 million years ago – the increasing levels of oxygen in the environment enabled animals to start evolving into the diverse life-forms that inhabit the Earth today (Lyons et al., 2014). Energetically speaking, life with oxygen is better than life without: a given amount of glucose processed in the presence of oxygen produces 18 times as much energy as the same amount of glucose processed without oxygen. Nevertheless, the use of oxygen also poses risks, and fluctuations in oxygen levels can lead to some of the toxic byproducts of metabolism accumulating in cells. Hence, animals have evolved elegant mechanisms to monitor oxygen levels and respond to changes.

In all animals studied so far, a specific set of molecules known as the HIF pathway has a central role in these mechanisms. When oxygen levels are low, this pathway – named after a protein called the hypoxia-inducible factor – triggers changes that help to maintain vital physiological functions in the cell (Bishop and Ratcliffe, 2014). Under normal oxygen conditions, an enzyme called prolyl hydroxylase modifies a particular amino acid (proline) in the HIF protein, which initiates its degradation via a protein called the von Hippel-Lindau protein. However, when oxygen levels are low, HIF remains intact and activates genes involved in anaerobic metabolism or processes that support oxygen delivery.

The HIF pathway has been found in the phylum Placozoa, which are among the simplest of all animal phyla (Loenarz et al., 2011), but until now it has not been clear if the ability to sense oxygen was present in the earliest animals, or if it evolved later. Now, in eLife, Gert Wörheide and colleagues at the Ludwig-Maximillians-Universität München and the University of Southern Denmark – including Daniel Mills and Warren Francis as joint first authors – report that early animals could have thrived without this pathway and survived with just a hint of oxygen (Mills et al., 2018).

The researchers decided to compare the first two lineages to have split from all other animals: sponges and comb jellies (Figure 1). Both phyla seem to survive extended periods of low oxygen, whereas the placozoans do not (Mills et al., 2014; Purcell et al., 2001; Loenarz et al., 2011). And since the oxygen levels in the ocean only increased about 200 million years after the first animals started to diverge, it is possible that animals had already started to evolve during a time when oxygen was scarce (Lyons et al., 2014; Dohrmann and Wörheide, 2017; Parfrey et al., 2011).

**Figure 1.**
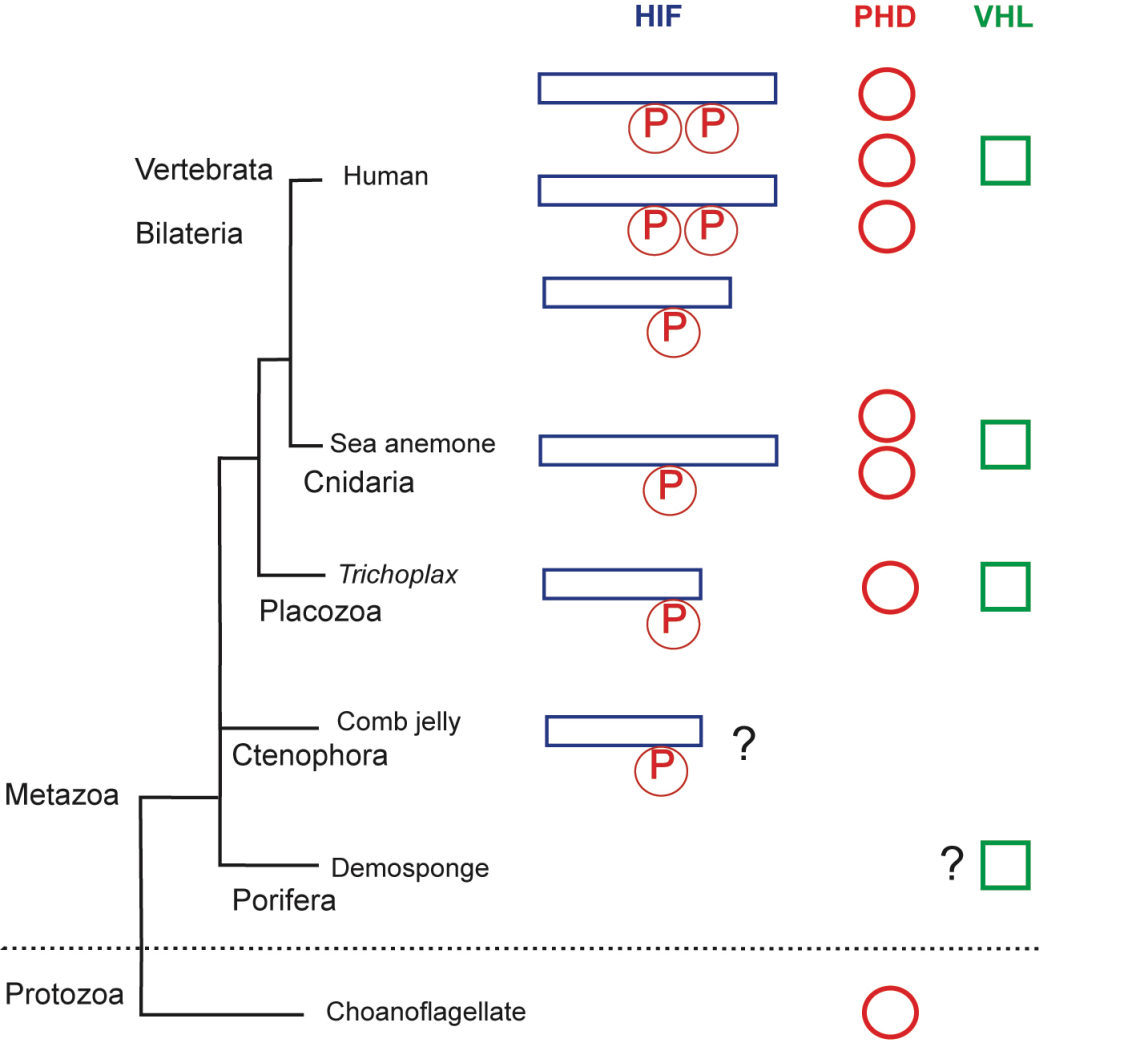
The evolution of the hypoxia-inducible factor (HIF) pathway. Animals probably began to diverge from a common ancestor around 800 million years ago, when oxygen levels were still scarce. Modern animals use a set of molecules called the HIF (hypoxia-inducible factor) pathway, the enzyme prolyl hydroxylase (PHD) and the von Hippel-Lindau protein (VHL) to regulate gene activity in the cells when oxygen is limited. The HIF protein contains a proline motif (P) that is targeted by PHD. It is unclear when the HIF pathway evolved. Choanoflagellates are simple, single-celled Protozoans that live in aquatic environments. They are the closest known relatives of animals and may be an evolutionary link to early animal life. Choanoflagellates do have PHD, but lack all other components of the HIF pathway. The sponges (Porifera) and comb jellies (Ctenophora) are the earliest animals to have split from a common animal ancestor, but it is debated which diverged first (King and Rokas, 2017). The sponges and comb jellies analyzed by Mills et al. lack key components of the HIF pathway, which suggests that the HIF pathway was not established in the common ancestor of modern animals (Metazoa), and can first be found in the Placozoan *Trichoplax adhaerens*. However, one comb jelly (*Eulopkamis dunlapae*) has an HIF with a proline motif that potentially could be targeted by PHDs (indicated by ?), but lacks PHD and VHL. For simplicity, Vertebrata (Human) are shown, but other bilaterian lineages are omitted. Two whole genome duplications in early vertebrates may be responsible for the multiple HIF and PHD genes found in human genome.

By comparing the genomes of several species of sponges and comb jellies, Mills et al. were able to show that they lack key gene components of the HIF pathway. This suggests that this pathway evolved after the last common ancestor of all living animals split from sponges and comb jellies and, therefore, it is not a universal trait of all modern animals.

Mills et al. make a convincing case for the absence of the HIF pathway in sponges: every sponge lacked most of the key components of the pathway. However, one of the comb jellies had a proline sequence in the HIF that was similar to sequences found in other animals. This could mean that the ancestors of comb jellies may have had an HIF-like pathway, but subsequently lost it at some point during evolution. The order in which the sponges and the comb jellies diverged from the other animals is currently debated (Feuda et al., 2017; King and Rokas, 2017). Evidence from anatomical and phylogenetic studies suggests that the sponges split first. However, if the comb jellies diverged first, it would challenge the idea that HIF was not present in the earliest animals.

Next, Mills et al. tested if the lack of an HIF pathway also led to the absence of (transcriptional) changes in gene activity when oxygen was scarce. When the sponge *Tethya wilhelma* was exposed to very low levels of oxygen (just 0.25% of modern levels), only a few genes changed over the course of four days of observation. Moreover, genes related to metabolism and stress did not change. However, when *T. wilhelma* was completely deprived of oxygen for just one hour, thousands of genes were activated, including several metabolic genes and a number of genes related to stress.

So, how can sponges respond to low oxygen conditions if they lack a functional HIF pathway? This pathway regulates the transcription of genes to make messenger RNA (mRNA), but it does not directly regulate the translation of mRNA to make proteins. There are, however, several oxygen dependent mechanisms that sponges could use to regulate translation: some bacteria, for example, use prolyl hydroxylases to target the components that produce proteins, while humans use specified gene duplicates to initiate protein production (Scotti et al., 2014; Uniacke et al., 2012).

Alternatively, sponges might use a different approach to respond to low levels of oxygen. One classic role of the HIF pathway is to redirect energy production away from aerobic reactions inside the mitochondia to anaerobic reactions taking place in the cytoplasm outside mitochondia. Mills et al. suggest that early animals could have responded to low levels of oxygen inside the mitochondria indirectly with the help of sulfide, which increases as oxygen diminishes. Sulfide is removed by oxygen-dependent enzymes, which are blocked when oxygen is low, and Mills et al. show that these enzymes are conserved in all sponges, comb jellies and all other clades of animals.

On a more fundamental level, we do not know if the earliest animals were ever exposed to oxygen or needed to respond to changes in oxygen levels. However, a better knowledge of mitochondrial metabolism in sponges could provide more clues, since mitochondrial energy production is not solely driven by oxygen (Müller et al., 2012). Some modern animals, such as intracellular parasites and animals living in tidal zones or sulfide-rich environments, can switch from aerobic to anaerobic energy production in the mitochondria rather than in the cytoplasm, in order to deal with lack of oxygen. Looking ahead, it will be interesting to see if *T. wilhelma* or some other member of the sponge phylum (which contains over 8500 species; Van Soest et al., 2012) are indeed capable of anaerobic energy production in the mitochondria.
